# Qualitative evaluation of trauma delays in road traffic injury patients in Maringá, Brazil

**DOI:** 10.1186/s12913-017-2762-6

**Published:** 2017-12-02

**Authors:** Anjni Patel, João Ricardo Nickenig Vissoci, Michael Hocker, Enio Molina, Nelly Moraes Gil, Catherine Staton

**Affiliations:** 1 Department of Surgery, Division of Emergency Medicine, Duke University, Durham, NC USA; 2 Department of Medicine, Faculdade Inga, Maringá, Parana Brazil; 3 Department of Emergency Medicine, Section of Prehospital and Disaster Medicine, Emory University, Atlanta, Georgia USA; 4 Department of Emergency Medicine, Augusta University, Augusta, GA USA

## Abstract

**Background:**

Road traffic injuries (RTIs) are the eighth leading cause of death worldwide, with an estimated 90% of RTIs occurring in low- and middle-income countries (LMICs) like Brazil. There has been minimal research in evaluation of delays in transport of RTI patients to trauma centers in LMICs. The objective of this study is to determine specific causes of delays in prehospital transport of road traffic injury patients to designated trauma centers in Maringá, Brazil.

**Methods:**

A qualitative method was used based on the Consolidated Criteria for Reporting Qualitative Research (COREQ) approach. Eleven health care providers employed at prehospital or hospital settings were interviewed with questions specific to delays in care for RTI patients. A thematic analysis was conducted.

**Results:**

Responses to primary causes of delay in treatment to RTI patients fell into the following categories: 1) lack of public education, 2) traffic, 3) insufficient personnel/ambulances, 4) bureaucracy, and 5) poor location of stations. Suggestions for improvement in delays fell into the categories of 1) need for centralized station/avoid traffic, 2) improving public education, 3) Increase personnel, 4) increase ambulances, 5) proper extrication/rapid treatment.

**Conclusion:**

Our study found varied responses between hospital and SAMU providers regarding specific causes of delay for RTI patients; SAMU providers cited primarily traffic, bureaucracy, and poor location as primary factors while hospital employees focused more on public health aspects. These results mirror prehospital system challenges in other developing countries, but also provide solutions for improvement with better infrastructure and public health campaigns.

## Background

Worldwide, over 1.2 million people are killed each year in road traffic injuries (RTIs) [1]. RTIs are currently the 8th leading cause of death globally, and are projected to be the 5th leading cause of death by 2030 [1]. Of concern, 90% of RTIs occur in low- and middle-income countries [1]. While RTIs are decreasing in high income countries (HIC) secondary to increased focus on safety and prevention, they are increasing at a disproportionately high rate in low- and middle-income countries (LMIC) due to economic growth and increased road traffic. In Brazil, more than 43,800 people are killed in road traffic crashes annually with 52% of deaths attributed to motorcyclists, bicyclists or pedestrians [1]. Paraná state in Southern Brazil has the 3rd highest number of road traffic-related deaths in the country; the city of Maringá had the second highest number of traffic accidents in the state in 2014 [2].

Road traffic injury victims in LMIC have a more significant burden of morbidity and mortality [3]. In countries with limited or developing prehospital care systems, delays in presentation to emergency departments or tertiary care centers likely contribute to overall morbidity and mortality [4]. The majority of trauma deaths, particularly in developing countries, occur in the prehospital setting [4]. Emergency physicians and trauma surgeons are classically taught that trauma patients presenting within the “golden hour”, the first hour after an injury, are associated with better outcomes. Although there is some controversy regarding the dogma of the “golden hour” with no specific time interval associated with improved outcomes studies have demonstrated decreased mortality with decreased time to presentation [5–10]. Prehospital Trauma Life Support training also stresses the importance of rapid transport to a trauma center for definitive treatment after initial stabilization [11]. Data from LMIC demonstrate limited or delayed prehospital care with the majority of deaths occurring prior to hospital presentation [4, 12, 13]. An approximated 24 million deaths per year in LMICs result from conditions sensitive to prehospital/emergency care [14]. This suggests that improvements in prehospital care can potentially decrease mortality from trauma patients [15–17]. Although trauma patients would specifically benefit from improved response and transport times, other time dependent diagnoses, such as STEMI (S-T Elevation Myocardial Infarction) and stroke, could also benefit. Much of the research to date on hospital presentation delays for trauma patients has been from HIC. There is minimal research in evaluation of the cause of delays in the transport of RTI patients to trauma centers in LMIC and even less data from Brazil. Therefore, the objective of this study is to determine specific causes of delays in prehospital transport of RTI patients to designated trauma centers in Maringá, Brazil.

## Methods

### Population and setting

This research was conducted in the state of Paraná at two sites: Metropolitano Hospital located in Maringá and the primary Maringá/Sarandí prehospital medicine station located in Maringá near the boundary of Sarandí, a town bordering Maringá. The Metropolitano Hospital is one of 3 state-designated regional trauma centers in the area. It is a public hospital with an Emergency Care Unit, Intensive Care Unit and neurosurgical, orthopedic and general surgery capabilities available 24 h per day, 7 days per week. The Emergency Care Unit maintains 5 acute care beds and 2 observation beds and is staffed at all times by at least 1 physician and 2 nurses.

The Brazilian prehospital system, Serviços de Atendimento Móvel de Urgência (SAMU), was implemented in 2002 and uses the French model of emergency medical services, utilizing physicians on ambulances to provide more advanced care, a “stay and play” model, as opposed to “scoop and run” as seen in the United States [18]. The Maringá/Sarandí SAMU catchment area covers roughly 500,000 people with an estimated monthly call volume of 3000, half of which are inter-hospital or clinic transport. All calls to *192*, the national emergency hotline number, are routed to the SAMU Regulatory Center, located in Maringá. The initial call is answered by a non-physician operator who directs the call to a medical receiver (MR), a trained physician. The MR activates the advanced or basic life support team who is then dispatched by a second operator.

The current SAMU system maintains one Advanced Life Support (ALS) unit team and 4 Basic Life Support (BLS) teams. The ALS unit consists of a physician medical interventionist (MI), nurse practitioner, and driver in an ambulance with intensive care capabilities and equipment. Physicians require a minimum of 2 years of medical training in addition to 30 days of prehospital training and continued education. All physicians are required to train as an MR prior to becoming a MI on an ALS team. They maintain a broad range of practice and are able to perform many emergent procedures that are above the scope of practice of a paramedic in the United States, such as pericardiocentesis, surgical airway, thoracocentesis, and central line access. A recent study indicates that only a small percentage of physicians working with SAMU have completed SAMU-specific or advanced prehospital training and many feel uncomfortable with the prehospital environment in the areas of pediatric trauma and surgical procedures (Tallo 2014). All physicians that we interviewed completed Advanced Trauma Life Support training and all prehospital care providers, including nurses and drivers, have completed Pre-Hospital Trauma Life Support (PHTLS) training [19]. The BLS unit consists only of a driver and nurse technician. Additionally, there are 3 private ambulance companies in the area each with one advanced life support team. SAMU is the primary regulator for these companies and maintains the right to pull an advanced life support team from a private ambulance company if call volume exceeds their resources. Prior to the nation-wide implementation of SAMU, the fire brigade (*193*) traditionally responded to road traffic injuries [18]. Today, the fire brigade continues to work loosely with SAMU by responding to some road traffic crashes. However, the firemen have minimal medical training and mainly work to extricate and immobilize the patient.

Although there are helicopter emergency medical services (HEMS) available in Brazil and provided to the public via SAMU, at the time of this study there were only 3 HEMS stations in the state of Paraná. There are also private HEMS services available throughout the country. There were no HEMS resources located in the Maringá/Sarandí area at the time of this study.

### Ethics

All participants provided verbal and written consent to participate in this study. This study was approved by the Institutional Review Board at the Faculdade de Ingá, in Brazil, and by the Institutional Review Board at Duke University.

### Data collection

The data was collected over a two-week period in March 2015 and reported based on the Consolidated Criteria for Reporting Qualitative Research (COREQ) [20]. Our qualitative approach included interviews developed and conducted according to the phenomenological analysis methodological orientation. This orientation seeks to interpret the underlying meaning and perceptions about one’s (or a group’s) experience of given phenomenon [21]. Using this approach, we conducted interviews with healthcare professionals to comprehend their perception of the care of patients who were transferred from the scene of road traffic crashes to tertiary care centers. Participants were health professionals who work at either SAMU or Metropolitano Hospital.

Healthcare personnel were interviewed by 6 research assistants fluent in Portuguese (medical students), trained and supervised by the Brazilian authors (JRNV and NG). Both authors discussed the content of each interview before the next one was conducted. Inclusion criteria for subjects included healthcare professionals (physicians, nurses, nurse technicians, or ambulance drivers) working with either SAMU or the primary hospital for a minimum of 6 months in addition to expressing an interest in participating in the research project. Study participants did not know the principal investigator or research assistants beforehand and vice versa. The script for the interviews included two open ended questions followed by probing questions:

1. *In your opinion, what are the causes of delay in care and transfer of trauma patients from the scenes of road traffic crashes to the trauma center? Probing for why is that a cause of delay, how it impacts care, how the respondent felt about that cause and how the respondents handled that cause.*
2. *What measures do you think could reduce attendance and transfer of a trauma patient from from the scene to a trauma center? Probing for why that measure was not performed, what were the barriers to apply the measure and how they perceived it would impact care.*

### Participants

Participants were identified through convenience sampling from Metropolitano Hospital in Maringá and the primary Maringá/Sarandí EMS station. A total of seven SAMU providers were interviewed: one SAMU nurse (female), three SAMU physicians (all male), and three SAMU ambulance drivers (all male). The hospital providers included two nurses (both female) and two physicians (males). All participants that were approached agree to be interviewed.

### Interview procedures

All interviews were performed individually and occurred within hospital or SAMU facilities. Interviewers were all medical students, trained by the physician and PhD level researchers (AP, JRNV and NMG). All interviewers were female, in their third year of medical school and taking public health courses. Supervisors were male and a female public health professors at a medical school from Brazil (NMG and JRNV) and a female emergency medicine resident from the US (AP). The interview script was piloted with other medical students prior to collecting data. Research assistants conducted the interviews iteratively between interviewing, transcribing and initial coding. Iterations consisted of: (a) after each interview, the research assistants would transcribe the content; (b) then, the physician and nurse researchers (AP and NMG, respectively) would read the transcriptions and perform an initial coding individually; (c) afterwards, new training and discussion sessions were conducted with the research assistants to align the interview process and direct probing questions.

All interviews were recorded and on average lasted 10–15 min. The speakers were identified by SN1 (SAMU nurse), HN1, HN2 (Hospital nurses); HMD1, HMD2 (Hospital physicians); SMD1, SMD2, SMD3 (SAMU physicians), and SD1, SD2, SD3 (SAMU ambulance drivers). Interviews would start with a brief introduction and rapport establishing, followed by each of the starting questions listed previously, and followed up by probing questions. Probing questions included further explorations about key concepts within each response. Rapport was established by explaining the research goals and obtaining written informed consent.

All transcriptions were performed by the research assistants in less than 4 days from the original interview. Interviews continued until saturation was met at 11 interviews. All interviews were transcribed verbatim and translated to English by the research assistants who were all fluent in both Portuguese and English. Notes and impressions obtained during participant interviews were added to the original transcription as comments. All transcripts were reviewed for accuracy and were available to other participating researchers. All identifiers were removed from the transcript to ensure confidentiality.

### Data analysis

Following a phenomenological approach, we analyzed transcriptions according to three steps: (1) Interpreted a sense of the entire transcription through multiple readings; (2) Identified concept categories and extracted the ones that were relevant for the clinical reasoning process; and (3) Integrated different concept categories into emerging themes. Emerging themes were then articulated regarding their role in the reasoning process as a whole. We used an inductive approach with no pre-defined themes to guide the coding process.

### Coding

Coding was conducted using a thematic analysis approach. Two research authors (AP and NMG) initially coded each transcription, which was later cross-validated with interviewers and other participating researchers (CS and JRNV). All discrepancies were resolved through discussion. Data saturation, whereby no new or relevant emerging themes arose, was achieved by the time data had been collected from 11 participants. This research was carried out in Brazil and performed in Portuguese (the primary language of the site) thus quotations used for illustration in this report were back-translated from Portuguese to English and then from English to Portuguese by two independent translators and finally reviewed by a bilingual participating researcher (JRNV) to ensure translation accuracy. An initial coding session resulted a set of raw themes that were aggregated in second order themes presented in our results.

### Theme validation

The transcripts of individual interviews were sent to the participants for comments and alterations. Having participants read transcripts of their interviews is considered helpful in some qualitative studies as a method of quality control and validation. Participants can ensure that the transcript is a true record of what they intended to say or, where necessary, can elaborate or provide a more nuanced perspective.

## Results

A total of 11 health care providers were interviewed during a two-week period in March 2015. These included 4 Hospital Metropolitano employees (2 physicians and 1 nurse) and 7 SAMU employees (3 physicians, 1 nurse, and 3 ambulance drivers).

### Question 1

The participants’ responses about causes of delay in care and transfer of trauma patients to a trauma center yielded 12 codes, aggregated into 5 major themes: 1) lack of public education, 2) traffic, 3) insufficient personnel/ambulances, 4) bureaucracy, and 5) poor location of stations (Table 1). Traffic was the most common theme overall, cited by 6 SAMU employees and 1 hospital employee.

**Table 1:** Themes in response to question 1

Themes	Codes	Hospital employees	SAMU employees	Total
Traffic related issues	-High traffic volume -Wrong navigation information	1	6	7
Lack of public education	-Lack of traffic education -Lack of public education to respond to trauma -Lack of drivers awareness of ambulance right-of-way	2	1	3
Insufficient personnel	-Lack of personnel	2	1	3
Poor location	-Stations distance from crashes sites -Stations located far from important places of the city	0	3	3
Insufficient ambulances	-Lack of equipment in the ambulances -Not enough ambulances	1	1	2
Bureaucracy	-Long time to receive notification within the prehospital care system -Difficulty with patient admission	0	2	2

### Transport

Most participants cited that traffic was the largest barrier to care for trauma patients. “*The only factor is traffic. The amount of cars on the road hinder the passage of the ambulance...”* The density of cars as well as the traffic delays were cited but the principle of yielding or pulling over for an ambulance to pass is also limited in Maringa thus making meandering through traffic difficult. “*Brazil has poor education in traffic laws, by comparison, in the United States if you hear the an ambulance siren everyone gives way. Here it doesn’t happen, it doesn’t exist.* “This is also seen in this response: *“The Brazilian Traffic Code says that the driver of the ambulance must keep left of the road and the cars should go to the right...This isn’t what happens in Maringa or in other Brazilian cities. Drivers do not know where to go, and may even cause another accident. Sometimes ambulances do not have lights and sirens on and drivers move over for them to pass without need.”*

Another transport difficulty that was cited was the fact that the location of the SAMU station was very far from most of the calls which increased the time to reach the patient and thus delayed the time to prehospital provider. “*We have two SAMU bases in Maringa, one in the south and one in the north, and sometimes it is difficult to travel to a distant location” and “The first problem is the location of the SAMU base. I think the main delay is our path from here to the scene. For example, if we go to the other side of town, in the north of the city, with traffic it takes 15, 20 minutes. That is unacceptable.”*

### Bystander care

Generally, participants believed that trauma and multi-trauma events are chaotic, with an uninformed public about how to proceed and limited providers or police to manage the situation. *“People aren’t prepared to provide prehospital care on the street. No one knows what to do in a multiple trauma. People want to get up, others say it's not recommended. There is a lot of crowding and people are misinformed. People in general should be more empowered to know what to do at the time of trauma, because people are desperate, and nobody knows how to act.”*

### Insufficient personnel/ambulances

Some providers stated that delays in care stemmed from an overwhelming number of calls and a limited number of prehospital providers available as well as only one ALS Unit with one physician available at any time. *“Delays happen because there are only a few number of employees, and the demand is too heavy. This city has been growing over the last few years and I believe that the number of employees has not corresponded to this growth.”* Similarly, there was a concern that a mass casualty event with as few as 4 patients would shut down the entire prehospital system. *“If something happens that uses all our vehicles, that is 4 hours that Maringa and Sarandí be without doctors. This is absurd.”*

*“In Maringá we have four ambulances to almost 400,000 inhabitants, which is something completely impractical.” “Sometimes we…receive 6 or 7 calls in less than 2 minutes... we do not have enough vehicles to provide the necessary support.”*

### Bureaucracy

One participant brought up a unique challenge to care in Brazil. In order to obtain hospital based treatment in Brazil, while it is free, for certain injuries you must present to the appropriate hospital based on your date of birth. Therefore, prehospital providers have been forced to take a patient to a second hospital after arriving to a hospital and finding out the patient’s birthday. This bureaucratic challenge is exemplified in the following response. *“I think the biggest challenge is the acceptance of the patient at the destination. We sometimes have difficulty admitting the patient. The hospital often states that the case was not referred to them, that they have to follow guidelines. For example, you have to refer patients with fractures [to specific hospitals] according to their date of birth. For a patient who has a skull fracture or is obtunded and cannot speak you do not have this information.”*

### Question 2

When asked what specific measures could reduce delays in attendance and transfer of a trauma patient from a scene to the trauma center participant responses were categorized into 10 codes aggregated into the following major themes: 1) Need for centralized station/avoid traffic, 2) Improving public education, 3) Increase personnel, 4) increase ambulances, 5) proper extrication/rapid treatment (Table 2).

**Table 2:** Themes in response to question 2

Themes	Codes	Hospital employees	SAMU employees	Total
Need for centralized station/avoid traffic	- Need to avoid traffic - Need of a centralized station to control the care offer	2	1	3
Public education	- Education about ambulance in traffic - Education for onlookers that slow the process of care	2	3	5
Increase personnel	- More personnel to improve attendance speed - Need for more physicians to support patient care	1	2	3
Increase ambulances	- Increase the number of ambulances at disposal	1	0	1
Proper extrication/rapid treatment	- Provide proper and faster extrication - Decrease time from extrication and treatment - Improve timely access to patients	0	1	1

### Proper extrication and rapid treatment

Participants suggested that initial patient extrication and basic treatment measures could be improved. *“The basic life support team that goes to the site first...They could start volume replacement to improve mortality, in addition to reducing other damage, for example, hemorrhage control, reversing respiratory failure, and so on.”*

### Improving public education

Similarly, public health education in order to increase knowledge of basic first aid was cited as a very important factor that would reduce delays and improve outcomes. *“If people were more knowledgeable, for sure they would arrive in better condition to the hospital. An education campaign to let people know how to act in trauma scenes should be made - at least the basics topics.”* Public education was also noted for road use and how to let an ambulance pass through traffic. *“Educate the people, because what makes us late is the people in our way that don’t know what to do.” “In other countries, people hear the sound of the siren and immediately open a passage to let the ambulance pass. This is what I see in the movies. Here it is chaos.”*

### Increase in personnel and ambulances

Numerous participants suggested that there should be an increase in the number of health providers as well as ambulances. *“In my opinion, is necessary to add more professionals and increase the number of ambulances for a faster service.”* One participant suggested the occasional high burden of calls seen would likely suggest more ambulances are needed to cover this area. *“The problem is that sometimes the demand is too much, and we can’t meet all calls in the desired time. For example, sometimes we have 30 to 40 minutes with no occurrence and sometimes we receive 6 or 7 calls in less than 2 minutes.”*

### Centralized SAMU Station

Participants suggested that given the distance of the two SAMU stations, identifying a location that is more centralized would decrease the time to reach the patient.

## Discussion

Rapid economic growth and increased road traffic are primary factors leading to the overall increased rates of RTIs in LMICs. Responses from the providers highlight the burden of increasing road traffic in not only contributing to RTIs, but also as a significant cause of treatment delays in these patients. The current study demonstrates different responses from both hospital and SAMU providers regarding specific causes of delay for RTI patients with SAMU providers citing primarily traffic and transport challenges, limited healthcare personnel and ambulances, and education as primary factors in delays.

Nearly all SAMU providers in this study cited traffic as a primary cause of delay. Similar problems with traffic and need for more resources are seen in other LMICs such as Iran, where Bidgoli et al. found shortages of professional staff, ambulances, and dispatch sites as important barriers to providing effective pre-hospital care [15]. Compounding traffic delays is the fact that the SAMU station is poorly located and far from most calls; 3 SAMU providers cite its location as a cause in delay with one provider suggesting a more centralized base to improve transport times. Arreola-Risa et al. demonstrated the creation of additional bases as a low-cost improvement in prehospital transit times in Monterrey, Mexico [22]. A prior study by Mock et al. found a considerable discrepancy in the number of ambulance dispatch sites between 3 systems in countries of significantly different socioeconomic status. Not surprisingly, better infrastructure and a higher ratio of ambulance dispatch sites per population ratio resulted in improved transit times [4]. Geographic distribution of ambulance bases is an important contributor to response times. In the current study, the system has one ambulance dispatch site in the north of the city and one to the south, with the site in the south being the only one with an ALS team and physician. If an ALS team was needed in the north of the city, the team would need to travel through the middle of the city, passing through crowded streets and adding several minutes to transit time. Expanding the current system to a more tiered system with a centrally-based dispatch center with the ALS unit and a wider distribution of BLS units could also improve transit times [23]. In resource limited settings without funding to create additional bases, careful planning of initial placement of ambulance dispatch sites to more centralized locations could potentially have a significant effect on prehospital times.

Prior studies have demonstrated that for a scene distance greater than 10 miles from the hospital, air transport is faster than ground transport for hospital arrival time [24]. A recent Cochrane review demonstrated improved survival in HEMS transport compared to ground transport [25]. With a metropolitan region of nearly 6000 km^2^ in the Maringá/Sarandí catchment area, HEMS would appear to be a reasonable consideration. However, HEMS is resource-intensive, cost-heavy and as a result, is primarily implemented in developed countries. For countries or regions with limited basic trauma system care, further development of ground transportation systems is more likely to be of life-saving benefit than HEMS systems. In the setting of this study, increasing the current number of ground emergency medical service units may prove a more judicious use of funding before HEMS is implemented.

Based on our research, increases in prehospital resources and personnel are not keeping up with pace of population growth and development in this setting. After implementation of SAMU in 2002, a rapid growth in the number of SAMU was seen between January 2004 and July 2009 but has slowed since that time, suggesting a lack of further SAMU development despite growth of the population. The National recommendation for ALS units in Brazil is 1 per 450 thousand people, but the average in many states, including Paraná, is below this [26]. The Maringa/Parana region has a population of approximately 500,000, with only one ALS unit available at all times. Further contributing to physician shortages, there is considerable reluctance by physicians in Brazil to work on ambulances secondary to violent conditions and low pay [27].

In addition to SAMU ambulances, private ambulance companies and the fire department both respond to emergency calls. A study by Gonsaga compared response rates and mortality rates of SAMU and fire department calls for trauma patients. Although there was no difference in mortality rates, response times for the fire department were faster, and the fire department also responded to more traumas than SAMU [28]. In Porto Allegre, a large metropolitan city south of Maringá, ambulance systems currently run by the fire department and military police are being absorbed into SAMU and most calls routed through a single dispatch center [29]. A similar initiative in Maringá in addition to further training for firefighters in basic first aid and stabilization could not only improve coordination between the two different services, but also effectively increase the number of units and result in a wider distribution of units in use.

The providers in the current study commented both on lack of basic first aid knowledge in the general population, lay people crowding the scene and limited adherence to road traffic laws prohibiting ambulance passing. Suggested solutions for this specific problem were increasing public education and awareness. Bidgoli et al., observed similar negative perceptions of laypeople on scene by emergency medical service providers in Iran [15]. Formal public education campaigns could provide valuable information to the public regarding the appropriate actions to take in trauma situations. Information distribution in the form of ads, billboards and television commercials could reach large audiences. Targeting specific populations of individuals such as high school students, military personnel, taxi drivers and police officers could offer improvements in care and limit crowding after RTIs. Several countries have demonstrated effective training to laypeople to provide prehospital care with basic first aid training [30]. Training lay people in basic life support in mine and war zones in Iraq demonstrated improvement in mortality from 15.6% to 9.8% [31]. Jayaraman et al. demonstrated that these types of skills offered to police officers, taxi drivers, and community leaders were retained for up to 6 months without a significant burden of cost [32]. Although most of these studies occurred in countries that lacked formal EMS systems, there may be a role for similar training in other LMIC countries with less robust EMS systems and limited resources.

In our study, the hospital employees focused more on lack of public education and personnel, compared to the prehospital personnel focusing on traffic, poorly located SAMU locations as well as bureaucratic issues thus highlighting the different perspectives. Other studies of prehospital systems in LMIC countries have highlighted the lack of informal training of providers [15, 22, 33, 34]. In this setting, all SAMU providers are adequately trained with PHTLS, standards that are comparable to the rest of the country [29]. Obtaining additional input from other services contributing to overall prehospital care, such as the private ambulance services and the fire department may offer a more complete picture of the scenario. Additionally, understanding the perspective of the layperson during a trauma could provide additional understanding of how to target public health campaigns.

### Limitations

Although the COREQ model was used to ensure reliability and accuracy of data collection, qualitative data, data collection was conducted by medical students in Portuguese. As follow up questions were guided by the student rather than advanced prehospital trained providers could have limited some qualitative responses. Alternatively, US based prehospital trained providers (PI) would have had some contextualized limitations to conducting this qualitative assessment given the complex nature of Brazil’s prehospital system. Additionally, we were unable to return to the SAMU and Metropolitano hospital providers for them to review and verify the interview transcripts.

The study also focused only on the views of SAMU providers from the main ambulance dispatch center and only one of three trauma hospitals in the catchment area. Collecting data from other parties involved in the prehospital trauma care of the region, such as employees from the other dispatch center, other trauma centers, private ambulance companies or the fire department could have yielded additional information for a more complete picture of the system.

## Conclusion

In Brazil, the majority of SAMU providers are well trained and the prehospital system utilizes physicians for advanced care. The information collected through these surveys corresponds with challenges faced by many prehospital systems in other LMIC countries, particularly with respect to increases in road traffic and transport challenges, increased population growth combined with lack of resources and poor distribution of ambulance services and the need for further public education. Next steps could involve targeted public health campaigns and improvements in infrastructure and planning to improve overall care for RTI patients.

## Abbreviations

ALS: Advanced Life Support
BLS: Basic Life Support
COREQ: Consolidated Criteria for Reporting Qualitative Research
HEMS: Helicopter emergency medical services
HIC: High Income Country
LMIC: low- and middle-income country
MR: Medical Receiver
PHTLS: Pre-Hospital Trauma Life Support
RTI: road traffic injury
SAMU: Serviços de Atendimento Móvel de Urgência
